# Life history factors, personality and the social clustering of sexual experience in adolescents

**DOI:** 10.1098/rsos.160257

**Published:** 2016-10-05

**Authors:** Abram J. van Leeuwen, Ruth Mace

**Affiliations:** Department of Anthropology, University College London, London, UK

**Keywords:** adolescent sexual behaviour, life history theory, personality, social clustering, multiple classification models, Avon Longitudinal Study of Parents and Children (ALSPAC)

## Abstract

Adolescent sexual behaviour may show clustering in neighbourhoods, schools and friendship networks. This study aims to assess how experience with sexual intercourse clusters across the social world of adolescents and whether predictors implicated by life history theory or personality traits can account for its between-individual variation and social patterning. Using data on 2877 adolescents from the Avon Longitudinal Study of Parents and Children, we ran logistic multiple classification models to assess the clustering of sexual experience by approximately 17.5 years in schools, neighbourhoods and friendship networks. We examined how much clustering at particular levels could be accounted for by life history predictors and Big Five personality factors. Sexual experience exhibited substantial clustering in friendship networks, while clustering at the level of schools and neighbourhoods was minimal, suggesting a limited role for socio-ecological influences at those levels. While life history predictors did account for some variation in sexual experience, they did not explain clustering in friendship networks. Personality, especially extraversion, explained about a quarter of friends' similarity. After accounting for life history factors and personality, substantial unexplained similarity among friends remained, which may reflect a tendency to associate with similar individuals or the social transmission of behavioural norms.

## 1. Introduction

Adolescents in industrial societies like the United Kingdom vary substantially in their age at first sexual intercourse and other aspects of their sex lives [1]. Such behavioural variation between individual teenagers may cluster at higher levels of the social structure, i.e. members of particular social groups, such as friendship networks and neighbourhoods, may be more similar to each other than random members of the wider reference population. In order to fully understand behavioural variation in the real world, one needs to account for variation at individual and higher social levels. The evolutionary behavioural sciences suggest several approaches to explaining differences between teenagers in age-specific levels of sexual experience. Most prominently, it has been argued that humans adjust their life history pace to environmental features [2]. Additionally, the so-called Big Five personality traits [3,4], which have been argued to possibly reflect alternative life history strategies [5,6], may affect adolescents' sexual behaviour.

### 1.1. Life history theory and adolescent sexual behaviour

Life history theory addresses how organisms allocate resources to life's key activities of growth, development, maintenance and reproduction and attempts to explain variation in life history traits, such as age at sexual maturity, both between and within species, guided by the principle of inclusive fitness maximization [7,8]. Theoretically, environments characterized by high levels of extrinsic mortality—sometimes called ‘harsh’ environments—favour faster life histories, characterized by earlier reproduction and higher fertility (among other things), because this increases the likelihood of at least some reproductive success in the face of a high mortality risk. By contrast, low-mortality environments favour slower life history strategies because investments in longevity and offspring quality are more likely to pay off [9,10]. This is thought to explain, for example, why mortality rates are inversely correlated with age at first reproduction across mammalian species [11]; or why there is a strong positive association, at the country level, between life expectancy at birth and women's age at first birth [12].

Considering timing of sexual debut as an indicator of life history pace, cues of local mortality rates may be able to predict whether adolescents are sexually experienced or not. In this context, father absence, low parental investment, a stressful home environment, and household and neighbourhood socioeconomic deprivation have all been implicated as potential accelerators of sexual behavioural development [2,13–15]. Aside from the possibility that ‘childhood’ adversity effectively signals the state of one's wider socio-ecological context, growing up in an unfavourable environment may also have direct negative effects on an individual's health and thereby life expectancy, which similarly would favour early reproduction [16].

#### 1.1.1. Father absence and parental care

Father absence has received a lot of attention as a possible influence on life history pace. Girls who grow up in fatherless households tend to experience menarche at a slightly younger age [17–28]. Most relevant to this study, father absence has repeatedly been found to predict an early sexual debut relative to age peers [17,29–32], as well as a higher probability of teenage pregnancy [32,33]. Parent–child closeness fairly consistently predicts later first sexual intercourse, fewer sexual partners and positive patterns of contraceptive use (reviewed in [34]).

#### 1.1.2. Socioeconomic status

Numerous studies have reported a positive association between low household socioeconomic status (SES) and the likelihood of experience with sexual intercourse in adolescents [35]. Studies have found an inverse relationship between adolescents' educational expectations and achievements and their likelihood of having had sex [36–38]. In line with life history theory, this may be due to associations between SES, educational qualifications, and mortality and morbidity [39]. Alternatively, in societies with skill-based economies and competitive labour markets, investments in education (embodied capital) that make individuals more successful at resource acquisition may be incompatible with early reproduction.

#### 1.1.3. Neighbourhood deprivation

Neighbourhoods may present residents with morbidity and mortality cues. In line with life history predictions, young people from poorer, more crime-ridden and perceived-as-dangerous neighbourhoods have been reported to start having sex at earlier ages, be less likely to use contraception and, if female, more likely to become pregnant [34]. Lower neighbourhood-level life expectancy predicts earlier female reproduction [2,40,41]. Such neighbourhood effects could also be due to neighbourhoods functioning as resource environments marked by differing levels of opportunity for social advancement.

### 1.2. Personality

Personality, which has a strong genetic basis in humans [42], is one factor that appears to be key to understanding between-individual behavioural variation in humans and other animals [43]. Personality refers to individual behavioural dispositions that exhibit substantial consistency across time and situations [44,45].

The Five Factor Model [3,4] identifies five broad dimensions of personality (the ‘Big Five’). *Openness*, a.k.a. intellect/imagination, measures intellectual curiosity, behavioural flexibility and openness to new experiences. *Conscientiousness* refers to a tendency to be organized and a preference for planning over spontaneity. *Extraversion* covers such traits as sociability and assertiveness. *Agreeableness* is a disposition to trustfulness and friendliness toward others. Finally, more *emotionally stable* (=less *neurotic*) individuals have a lower tendency to experience psychological distress.

The evolution and maintenance of personality differences in humans and other animals have recently become a focus of evolutionary theorizing and modelling [46,47] and spawned a sizeable body of empirical research. Several models explain the evolution and maintenance of personality differences in terms of balancing selection [48]. Different personalities may represent alternative, but in the long run equally fit, life history strategies [5,6].

Nettle [5] suggested a scheme in which each of the Big Five traits represents a different evolutionary trade-off. In this scheme, *extraversion* represents a life history strategy that emphasizes mating effort and exploratory and social behaviour at the cost of increased exposure to risk. *Conscientiousness* is part of a strategy emphasizing long-term investments. Indeed, conscientiousness is associated with healthy behavioural habits, which actually result in increased longevity [49]. However, forgoing short-term rewards may be costly. *Agreeable* individuals benefit from potential cooperation partners, but may be more vulnerable to exploitation. There is ample evidence that more agreeable individuals tend to have higher-quality social relationships [50,51]. *Neuroticism* may have provided fitness benefits in ancestral environments because of its association with vigilance. It is also associated with competitiveness, which may translate into fitness benefits, given the right environment and competitive ability of the neurotic individual. On the cost side, highly neurotic individuals are more likely to suffer from a range of psychiatric disorders [52]. Finally, individuals high in *openness* may gain benefits from being creative but may also have an increased risk of psychotic disorder.

Personality differences between young people are associated with differences in sexual and reproductive behaviour. Extraversion has been shown to be positively associated with risky sexual behaviour and a higher likelihood of having had sex [5,53–55] and extraverted males also appear to have higher mating and reproductive success [55,56]. Past research has found young people with higher scores for conscientiousness, agreeableness and emotional stability to be less likely to have had sex [53].

Thus, if personality traits cluster for some reason, such as preferential assortment, then clustering of sexual behaviour could arise as a by-product. This possibility is all the more pertinent because a tendency to become friends with others with similar personality traits has been demonstrated in this study population, in particular, similarity in extraversion appeared to play a role in friendship formation [57]. A recent longitudinal study of emerging friendship networks showed that first-year university students were more likely to form friendships if more similar in (self-assessed) openness, extraversion and agreeableness [58] (but see [59], which only found an effect of *dissimilarity* in agreeableness among early adolescents). Adolescents may also be more likely to form friendships with others if they have a more similar level of intention to have sex [59].

### 1.3. Social structure and social clustering of behaviour

Social structure (e.g. neighbourhood residence) may cause a violation of the assumption of non-independence of data points. Rather than simply a nuisance to be taken into account for statistical-technical reasons, such dependencies can actually be very informative [60]. Multilevel models allow one to quantify behavioural variance at different levels of the social structure, i.e. social clustering of behaviour, which can suggest whether and where social-environmental influences might be found. They also allow one to estimate how outcome variance associated with a particular level changes in response to the addition of predictor variables, which may provide additional insights into reasons for clustering.

This approach is consistent with a multisystem or ecological approach, which recognizes that individuals occupy multiple micro- and macro-contexts (families, schools, peer groups, neighbourhoods, television and film, the Internet), which need to be studied in conjunction to gain a fuller understanding of contextual influences on behaviour [61]. Across the few studies that have taken a multisystem approach to the study of the antecedents of adolescent sexual behaviour by incorporating influences from three or more social contexts simultaneously [62–64], (risky) sexual behaviour was significantly associated with predictors from all contexts, demonstrating the potential importance of considering multiple social contexts.

#### 1.3.1. Friends

Numerous studies have confirmed that adolescents tend to be similar to their friends in terms of a range of attitudes, beliefs and behaviour [65,66]. Similarity of close associates has also been reported for other species [67–69]. At a proximate level, this may be partly due to individuals preferring to form and maintain social ties with similar others, a phenomenon often referred to as ‘similarity attraction’ [70]. A popular adaptationist explanation for why a preference for similarity evolved is because it facilitates coordination among collaborators engaged in task performance [71,72], although empirical work testing for such benefits is rare (but see [73]). Similarity of collaborative partners can also be the result, for modifiable traits, of changing trait values to fit with those of one's interaction partners. Thus, coordination benefits may also provide an evolutionary rationale for being susceptible to some forms of social influence [74].

A recent meta-analysis of the association between adolescent sexual behaviour and descriptive peer norms, that is, actual or perceived peer sexual behaviour, found a mean effect size, expressed as a correlation coefficient, of 0.40 [75]. Measures of sexual behaviour included experience with sexual intercourse, age at first intercourse, number of lifetime sexual partners, among others. Close friends appeared to be more similar (*r* = 0.45) than school peers (*r* = 0.29). Note that all but three of the 57 studies included in this meta-analysis used perceived rather than actual peer behaviour.

While friend similarities are often interpreted as evidence of peer effects, causality is hard to demonstrate because of alternative pathways to behavioural similarity, such as shared ecology and similarity-based assortment, that are often difficult to tell apart on the basis of observational data [76]. Nonetheless, studies using a range of different methodologies designed to identify such a causal link have suggested the existence of friend effects on adolescent sexual behaviour [77–80]. This study uses information on actual (self-reported) sexual activity of friends, that is, descriptive norms of peer behaviour, which a recent meta-analysis found to be more strongly associated with an adolescent's own sexual experience than either injunctive norms (i.e. peer (dis)approval) or peer pressure [75].

#### 1.3.2. Schools

Schools may affect sexual behaviour through a number of channels, including peer effects [78]. As children become adolescents, the role of the family as the child's dominant socializer shifts to the age peer group, which, in modern societies, tends to be heavily concentrated within a young person's school. A consensus on the existence and importance of school-peer effects is yet to emerge, possibly due to the methodological difficulties associated with differentiating between reasons for behavioural clustering in schools [81].

While few studies have explicitly quantified school-level clustering of adolescent sexual behaviour, a number of studies have found average school- or class-level sexual behaviour to be predictive of individual sexual behaviour [78,82]. Based on the school- and neighbourhood-level variances reported by Teitler & Weiss [83] across a large American city, the intra-school correlation for experience with sexual intercourse can be calculated as approximately 10% [83].

#### 1.3.3. Neighbourhoods

As discussed above, clustering of sexual experience in neighbourhoods may reflect neighbourhood-level morbidity and mortality cues or neighbourhoods' role as resource environments. But neighbourhoods could also influence the behaviour of adolescents as arenas for social transmission of behaviours, attitudes and beliefs. A recent study [84] suggests that the normative climate of a neighbourhood—operationalized as a neighbourhood-aggregated measure of sexual attitudes among adolescents—is associated with tendencies toward risky sexual behaviour (as indicated by early sex, casual sex, number of sexual partners), net of the impact of neighbourhood deprivation and demographic characteristics.

In the study by Warner *et al.* [84], a model with just the normative climate variable implied a neighbourhood intraclass correlation of 7.7% [84]. The model lacks a school level, however, which makes it vulnerable to misattribution of variance. By contrast, another study from the USA found neighbourhoods to be relatively unimportant when both schools and neighbourhoods were included in a cross-classified model looking at whether someone had ever had sexual intercourse [83]. Based on the reported variances, we have calculated the intra-neighbourhood correlation as 2.9%.

### 1.4. This study

In this study, we use data from the Avon Longitudinal Study of Parents and Children to investigate how much of the variation in experience with sexual intercourse by 17.5 years is found at different levels of the social world—individual and family, neighbourhood, school, and friendship networks—in a sample of British adolescents. Having revealed the pattern of clustering, we examine how much of it can be explained by similarity in a set of predictors believed to be important from a life history perspective as well as the Big Five personality factors.

## 2. Material and methods

### 2.1. Data and participants

The primary data source for this study was the Avon Longitudinal Study of Parents and Children (ALSPAC) [85,86]. ALSPAC is a large and ongoing birth cohort study centred on the city of Bristol (estimated population in 2015: 442 500) in the south-west of England. The original sample contained 14 451 pregnancies, with expected delivery dates between the 1 April 1991 and 31 December 1992, which resulted in 14 062 live births (13 988 children alive at 1 year of age). The study sample is broadly representative of the British population in terms of socioeconomic and medical markers although there is some overrepresentation of White, affluent Britons. Ethical approval for the study was obtained from the ALSPAC Ethics and Law Committee and the Local Research Ethics Committees. The ALSPAC website contains details of all the data that are available through a fully searchable data dictionary (http://www.bris.ac.uk/alspac/researchers/data-access/data-dictionary/).

Most of the data used in this study were collected through postal questionnaires sent to the study children's mothers every few months. Information on adolescent sexual behaviour was obtained in a computer session during their visit to the so-called Teen Focus 4 clinic (TF4; target age = 17.5). The Office for National Statistics produced the local deprivation data used below. ALSPAC has been matched to the UK government's National Pupil Database, which provided educational data (*viz*., an anonymized school identifier and educational achievement at the end of Key Stage 4).

Our initial study sample consisted of 4058 adolescents in the core ALSPAC sample with a valid outcome value (had sexual intercourse: yes/no). After excluding twins, individuals without a valid school or area identifier, and adolescents with missing values for more than 75% of predictors, we were left with an analysis sample of 2877 adolescents.

A comparison of this sample with the attrition sample—i.e. all individuals (singleton births) in the core ALSPAC sample alive at 1 year but not included in the analysis sample, revealed a skew of the analysis sample toward higher SES (see electronic supplementary material, S1). Adolescents in the analysis sample also tended to live in less deprived areas: only 2.8% of the study adolescents lived in the 10% most deprived areas in England.

### 2.2. Variables

#### 2.2.1. Dependent variables

At Teen Focus 4 (target age = 17.5), participants were asked a number of questions about their sexual history, including whether they had ever had sexual intercourse with another young person. They were not asked directly about their age at first sex.

For this age group, sexual activity can be considered normative behaviour in the population under consideration (British teenagers). According to figures from the third National Survey of Sexual Attitudes and Lifestyles (Natsal-3), the median age at first heterosexual intercourse in England, Scotland and Wales for the age group 16–24 years is 16 (IQR: 15–18) for both males and females [1].

#### 2.2.2. Independent variables

*Socioeconomic status*. Maternal and paternal education were included as categorical variables with five categories based on the highest educational qualification obtained. The categories, from low to high, are: (i) no qualification/CSE, (ii) vocational qualification, (iii) O-levels, (iv) A-levels and (v) university degree. A categorical variable indicated whether mothers reported experiencing no, some, or many financial difficulties when the study child was approximately 7 years old, based on reported difficulties in affording food, clothing, heating, rent or mortgage, ‘things you need for your children’, ‘costs of educational courses', medical or dental care, and child care. Home ownership status—renting, mortgaging or owning—was assessed when the study child was around 10 years old.

*Parental life history pace*. Parents act as behavioural models for their children and often make efforts to instil values they deem important and transmit norms of appropriate behaviour to their offspring. Variation in sexual behaviour, and the timing of puberty, may also have a genetic basis [87]. Thus, one might expect the adolescent offspring of parents on a faster life history trajectory to be more sexually experienced at an earlier age (e.g. [88]).

The analysis models included variables intended to capture parental life history pace. All of these were reported by the mother during the study pregnancy. The two most direct measures were maternal age at first pregnancy and whether the mother reported having voluntary sexual intercourse with another young person before the age of 16. We also included mother's reported age at menarche, as timing of puberty is considered a life history trait, which also predicts the onset of sexual behaviour [89], and paternal age at index pregnancy. Male first pregnancy data were not available.

*Parental investment and father absence before the age of 10*. The parenting scores used to examine the importance of paternal and maternal care (first derived in [90]) are standardized measures based on the frequency with which the mother reported engaging in a set of parenting activities involving her direct interaction with the study child (mother score) and the frequency with which the father was reported by the mother to perform the same set of activities. Example activities include playing with toys, bathing the child and putting the child to bed. The minimum score of 0 indicates that a parent did not engage in any of the parenting activities, while the maximum score of 10 means that he or she engaged in all of them at maximum frequency, that is, ‘nearly every day’. The parenting scores used here were assessed when the child was 18 months old.

We also examined the association between father absence before the age of 10 and adolescent sexual behaviour. If the biological father was present in the household at 10 years, adolescents were considered to have grown up in a father-present household; if the biological father was not present at 10 years, they were treated as having grown up in a father-absent household.

*Neighbourhood deprivation.* Our measure of neighbourhood deprivation was the Index of Multiple Deprivation 2010. Individual records in ALSPAC can be linked to indices of local deprivation produced by the UK government's Office for National Statistics. The English Indices of Deprivation 2010 (see [91] for details of construction) are measures of deprivation for so-called lower layer super output areas (LSOAs). LSOAs are geographical areas defined by the ONS for statistical purposes, with populations numbering between 1000 and 3000 (or 400 and 1200 households), designed to cover a relatively small geographical area and exhibit some degree of social homogeneity (using criteria related to housing).

The English Indices of Deprivation 2010 comprise indices of deprivation in seven domains plus a summary measure known as the Index of Multiple Deprivation 2010 (IMD 2010). The domain indices roughly correspond to the proportion of the population on a low income, involuntary unemployment, poor health outcomes, lack of educational achievement, access to essential services (e.g. primary school, GP) and housing, crime levels, and the quality of housing and the living environment. The IMD 2010 mainly uses data from 2008. Higher scores indicate more deprivation.

*Personality.* A 50-item questionnaire based on the International Personality Item Pool (IPIP) [92] was administered when participating adolescents were around 13.5 years old. The IPIP is based on the Five Factor Model. For the sample, Cronbach's *α* ranged between 0.72 for agreeableness and 0.85 for extraversion, indicating acceptable to good internal consistency for all scales.

*Adolescent's educational achievement*. We considered the educational achievement of participating adolescents at the end of Key Stage 4, when participants were generally around 16 years old. At this stage of their school careers, pupils in England typically complete a number of general certificates of secondary education (GCSEs). These are graded, from best to worst, A*, A, B, C, D, E, F, G or U (i.e. ‘ungraded’ or ‘unclassified’). The measure of educational achievement used here is the number of GCSEs graded A or A*.

*Pubertal development, age and sex*. A binary measure of pubertal development, based on information from a questionnaire administered when the study adolescents were about 13 years old, was included as earlier puberty has been found to predict an earlier sexual debut [93]. If a girl had started her menstrual periods or a boy's voice had changed, this was coded as 1 (versus 0 = no periods yet/no change in voice). Age at Teen Focus 4 was a covariate in all models. Finally, sex of the respondent was included to account for possible differences between girls and boys in sexual behavioural development.

#### 2.2.3. Social structure

*Friendship networks*. Friendship network assignments were based on a questionnaire called *You and Your Friends*, which ALSPAC participants were sent in 2008, when they were 15–17 years old, and which asked them to list up to five friends. This questionnaire was returned by 3123 participants who nominated 14 503 friends (11 041 unique individuals) [57]. Nominated friends' names were used to link them to ALSPAC; restricting the sample to ALSPAC participants only leaves 2396 respondents who listed 6961 friends (4572 unique individuals). We assigned individuals in our study sample to friendship networks based on an undirected edge list. An undirected edge list was used because, firstly, many ALSPAC-participating nominees did not complete the *Friends* questionnaire, making reciprocity impossible to ascertain, and, secondly, this maximized the number of ties included in the analyses. Two kinds of links between individuals were used: direct nomination links (A nominated B, vice versa, or both) and links though a third individual (A and B both have direct link to C). This procedure left 1115 individuals in 411 friendship networks with 2 or more members (rather than 446 individuals in 223 friendship networks if only reciprocated ties had been used). While some individuals belong to multiple friendship networks, we used only one, randomly chosen friendship network per respondent in the analyses that follow.

*Schools.* ALSPAC's anonymized secondary school identifier at Key Stage 4 (missing for approximately 18% of the core sample), when pupils are approximately 15 years old, acts as the school classification. Adolescents in the analysis sample attended 79 schools with a mean of 36.4 respondents per school (range: 1–204).

*Neighbourhoods.* The aforementioned lower super output areas functioned as the neighbourhood classification. The LSOA data indicate where respondents were living on 1 January 2008, when they were, on average, about 17 years old. The respondents in the study sample resided in 563 areas, with a mean of 5.1 respondents per neighbourhood (range: 1–18). Some residential mobility was evident in the sample: between 1 January 2001 and 1 January 2005, 457 of the 2,839 (16.1%) respondents for which the residential LSOA is known at both time points moved to a different LSOA in the Bristol area; between 1 January 2005 and 1 January 2008, 216 out of the 2868 (7.5%) respondents moved to a different LSOA in the Bristol area.

### 2.3. Analysis

#### 2.3.1. Modelling approach

We ran logistic multiple classification models [94] with individuals nested in schools, areas and friendship networks [95] to investigate the social clustering of sexual experience and its association with life history predictors.

First, we ran a set of models without predictors (bar age and sex) to assess the amount of variance located at different levels of the social structure, each model using a different combination of the three social structure classifications, from individual only to all classifications (making eight clustering-only models in total). While the full clustering-only model is of most interest, running the full set of social structure models may suggest ways in which models with a simpler structure lead to the misattribution of variation. For example, as friends often attend the same school, studies without information on friends, may erroneously attribute variation at the level of friendship networks to schools.

Next, we ran eight substantive models with different blocks of life history predictors: an SES model (maternal and paternal education, financial difficulties and home ownership status); a *parental life history pace* model (maternal ages at menarche and first birth, a binary variable indicating whether the adolescent's mother had had sexual intercourse before her 16th birthday, and lastly paternal age at index pregnancy); a *parental investment* model (female and male parenting scores and father absence before age 10); a *neighbourhood deprivation* model (IMD 2010); a *personality* model (Big Five personality factors); a *pubertal development* (pubertal timing measure); a model with all of the foregoing predictors; and, finally, a modified version of the full model which also included the participant's educational achievement. We did not include educational achievement in earlier models because it is arguably itself a life history trait and, moreover, strongly related to parental SES. Note that if adding a predictor leads to a reduction of the share of the total variance at a particular level of the social structure, it suggests that part of the similarity of members of the same group at that level is actually due to similarity of the added predictor [95].

The model estimates of the classification variance parameters from the logistic regressions were used to calculate residual intraclass correlation coefficients (ICCs) with a latent variable approach [96]. The ICC is a measure of the similarity of members of the same group, *viz.*, the expected correlation in the outcome of interest between two randomly selected members of the same group. All analyses used Markov Chain Monte Carlo (MCMC) estimation methods, as implemented in MLwiN [97]. Statistical modelling was performed in MLwiN 2.30 [98] run from within Stata 12 [99] using the command *runmlwin* [100].

Models can be compared on the basis of the deviance information criterion (DIC), a measure that combines model fit and complexity [101]. Some rules of thumb for the interpretation of information criteria are as follows: when the difference in DIC between a particular model and the best model in the candidate set (*lowest* DIC) is less than or equal to 2, the model in question has ‘substantial support’, a model whose DIC is between 4 and 7 points higher than the best has ‘considerably less support’, and a model with a DIC that is 10 or more points higher has ‘essentially no support’ [102].

#### 2.3.2. Missing data

The proportion of missing data varied between 19.4% for conscientiousness and 0% for sexual experience by approximately 17.5, sex, age and neighbourhood deprivation. For participants, the mean number of missing values in the final sample was 2.05, with a maximum of 14, although more than 80% of participants in the final sample had fewer than 5 missing values. We used multiple imputation, conducted in Stata 12, to avoid well-known problems associated with complete-case analysis (biased parameter estimates and loss of power) [103]. Prior to imputation, we standardized all age variables, neighbourhood deprivation, parenting scores and personality trait scores, and log-transformed neighbourhood deprivation, cubed the mother's parenting score, and squared the father's parenting score in order to approximate normal distributions. All analyses were performed on 20 imputed datasets. We used ‘mi estimate’ command in Stata 12 which calculates parameter estimates for each imputed dataset individually and then combines them according to Rubin's rules [104].

Data were more likely to be missing if collected later during the study. Family SES was negatively correlated with the proportion of missing data. For example, the mean number of missing values was 3.5 if mothers were in the lowest education category, which decreased with increasing educational level to 1.3 for the highest education category.

## 3. Results

### 3.1. Sample description

Descriptive statistics for the study variables are given in table 1. Their pairwise correlations can be found in electronic supplementary material, S2.

**Table 1.** Descriptive statistics for model variables.

		all		did not have sex	had sex
variables	units *or* categories	mean (s.d.) or distribution across categories (%)	*n* (% of sample)	mean (s.d.) or distribution across categories (%)	mean (s.d.) or distribution across categories (%)
had sexual intercourse by Teen Focus 4	no	1031 (35.8%)	2877 (100%)	1031 (100%)	0 (0%)
	yes	1846 (64.2%)		0 (100%)	1846 (100%)
age	years	17.75 (0.35); range = 16.42–19.42	2877 (100%)	17.69 (0.31)	17.78 (0.37)
sex	male	1244 (43.2%)	2877 (100%)	531 (51.5%)	713 (38.6%)
	female	1633 (56.8%)		500 (48.5%)	1133 (61.4%)
maternal education	none/CSE	350 (12.4%)	2826 (98.2%)	93 (9.2%)	257 (14.2%)
	vocational	230 (8.1%)		68 (6.7%)	162 (8.9%)
	O-levels	1050 (37.2%)		334 (33.0%)	716 (39.5%)
	A-levels	745 (26.4%)		293 (29.0%)	452 (24.9%)
	degree	451 (16.0%)		223 (22.1%)	228 (12.6%)
paternal education	none/CSE	509 (18.5%)	2754 (95.7%)	138 (13.9%)	371 (21.1%)
	vocational	235 (8.5%)		74 (7.4%)	161 (9.2%)
	O-levels	640 (23.2%)		222 (22.3%)	418 (23.8%)
	A-levels	786 (28.5%)		283 (28.4%)	503 (28.6%)
	degree	584 (21.2%)		279 (28.0%)	305 (17.4%)
financial difficulties	none	1272 (50.4%)	2522 (87.7%)	498 (53.6%)	774 (48.6%)
	some	964 (38.2%)		336 (36.2%)	628 (39.4%)
	many	286 (11.3%)		95 (10.2%)	191 (12.0%)
home ownership status	mortgaged	2110 (84.8%)	2487 (86.4%)	793 (85.7%)	1317 (84.1%)
	owned	176 (7.1%)		82 (8.9%)	94 (6.0%)
	rented	201 (8.1%)		50 (5.4%)	151 (9.7%)
mother had sex with boyfriend when less than 16	no	2154 (85.3%)	2526 (87.8%)	827 (90.0%)	1327 (82.6%)
	yes	372 (14.7%)		92 (10.0%)	280 (17.4%)
maternal age at first pregnancy	years	25.72 (4.81); range = 14–42	2845 (98.9%)	26.57 (4.71)	25.25 (4.80)
maternal age at menarche	years	12.83 (1.47); range = 8–22	2515 (87.4%)	12.89 (1.49)	12.80 (1.46)
paternal age at index pregnancy	years	31.42 (5.44); range = 16–60	2693 (93.6%)	31.95 (5.29)	31.12 (5.50)
female parental care at 18 months	10-point scale	8.01 (0.86)	2740 (95.2%)	8.01 (0.84)	8.02 (0.86)
male parental care at 18 months	10-point scale	6.18 (1.55)	2653 (92.2%)	6.23 (1.54)	6.16 (1.56)
father absence at 10 years	present	2187 (83.8%)	2610 (90.7%)	842 (88.5%)	1345 (81.1%)
	absent	423 (16.2%)		109 (11.5%)	314 (18.9%)
index of multiple deprivation	composite score	13.97 (11.45); range = 1.43–70.36	2877 (100%)	12.97 (10.80); range = 1.43–70.36	14.52 (11.76); range = 1.43–70.36
pubertal development: voice broken (m) or started menstrual periods (f)	no	884 (43.6%)	2026 (70.4%)	396 (52.2%)	488 (38.5%)
					
	yes	1142 (56.4%)		362 (47.8%)	780 (61.5%)
extraversion	IPIP score	35.15 (6.97)	2420 (84.1%)	32.60 (7.04)	36.60 (6.50)
agreeableness	IPIP score	38.38 (5.01)	2365 (82.2%)	38.12 (5.07)	38.52 (4.97)
conscientiousness	IPIP score	32.10 (5.65)	2318 (80.6%)	32.96 (5.66)	31.61 (5.58)
emotional stability	IPIP score	31.65 (6.49)	2346 (81.5%)	32.31 (6.24)	31.27 (6.60)
openness	IPIP score	36.26 (5.58)	2366 (82.2%)	36.49 (5.62)	36.13 (5.55)
adolescent education: GCSE results	no A or A* result	1179 (40.1%)	2877 (100%)	308 (29.9%)	871 (47.2%)
	1 A or A* result	382 (13.3%)		126 (12.2%)	256 (13.9%)
	2 A or A* results	228 (7.9%)		85 (8.2%)	143 (7.8%)
	3 A or A* results	170 (5.9%)		62 (6.0%)	108 (5.9%)
	4–6 A or A* results	393 (13.7%)		174 (16.9%)	219 (11.9%)
	7–13 A or A* results	525 (18.3%)		276 (26.8%)	249 (13.5%)

### 3.2. Clustering

The first set of models explored the clustering of sexual experience across the social structure, without including substantive predictors (apart from the covariates age and sex). Note that we compare clustering for all combinations of social classifications (one combination per model). These clustering-only models were therefore not performed in any particular order and do not involve model selection. Table 2 lists the clustering models in order of goodness of fit, starting with the best-fitting model according to the DIC. The school-only model (DIC = 3643.90), neighbourhood-only model (DIC = 3664.82) and friendship-network-only model (DIC = 3610.51) all fitted the data better than the individual-only model (DIC = 3672.43). However, inclusion of the neighbourhood classification alongside either the school or the friendship-network classification (or both) does not improve the model fit compared with the same models without neighbourhoods, which suggests that the latter were ‘borrowing’ variance from schools and/or friendship networks in the neighbourhood-only model. Inclusion of the friendship network had the largest impact on model fit. Models without both the friendship network and the school classification received essentially no support (ΔDIC ≥ 10). The model without the neighbourhood classification, however, received substantial support (ΔDIC ≤ 2), suggesting that the neighbourhood classification could be left out of the model without much of an impact on model fit.

**Table 2.** Social clustering models ranked by model fit, in descending order based on deviance information criterion.

classifications included	DIC	ΔDIC
individual + school + neighbourhood + friendship network	3585.97	0
individual + school + friendship network	3586.96	0.99
individual + friendship network	3610.51	24.54
individual + neighbourhood + friendship network	3610.81	24.84
individual + school + neighbourhood	3643.32	57.35
individual + school	3643.90	57.93
individual + neighbourhood	3664.82	78.85
individual	3672.43	86.46

Table 3 provides model estimates of the (residual) variance associated with the different classifications, for the single-classification models and the combined model, and expresses these as ICCs. Even in the single-classification models without substantive predictors (apart from respondent age and sex), the residual intraclass correlation at the level of schools (0.029) and neighbourhoods (0.033) was very modest. In the combined model containing all social classifications, a residual ICC of 0.023 was found for schools while only 0.010 remained for neighbourhoods. A far larger residual ICC was found for friendship networks: 0.25 in the combined model.

**Table 3.** The social clustering of adolescent sexual behaviour: residual variances and intraclass correlations at the school, neighbourhood and friendship-network level.

		individual	school	neighbourhood	friendship network	combined
classification						
school	variance		0.098			0.107
	rICC^a^		0.029			0.023
neighbourhood	variance			0.113		0.048
	rICC			0.033		0.010
friendship network	variance				1.248	1.146
	rICC				0.275	0.250
	DIC	3672.43	3643.90	3664.82	3610.51	3585.97

^a^rICC = residual intraclass correlation.

### 3.3. Life history predictors

#### 3.3.1. Unadjusted models

The results for the unadjusted SES, parental life history pace, parental investment, neighbourhood deprivation, personality and pubertal development models are given in table 4. When considering the explained variance, note that control variables age and sex explained about 4.2% of the variance when considered in isolation.

**Table 4.** Model results for unadjusted socioeconomic status, parental life history pace, parental investment, neighbourhood deprivation and personality model predicting whether adolescents have had sexual intercourse.

	socioeconomic status			parental life histories			parental investment			neighbourhood deprivation			personality			puberty		
parameter	OR	95% CI	*p*-value	OR	95% CI	*p*-value	OR	95% CI	*p*-value	OR	95% CI	*p*-value	OR	95% CI	*p*-value	OR	95% CI	*p*-value
age at Teen Focus 4	1.35	1.21–1.50	<0.001	1.35	1.21–1.49	<0.001	1.36	1.23–1.51	<0.001	1.36	1.23–1.51	<0.001	1.36	1.22–1.51	<0.001	1.35	1.22–1.50	<0.001
sex	0.55	0.45–0.67	<0.001	0.54	0.44–0.66	<0.001	0.53	0.44–0.65	<0.001	0.53	0.43–0.65	<0.001	0.68	0.54–0.85	0.001	0.57	0.47–0.70	<0.001
*maternal education (ref.: none/CSE)*																		
vocational	0.92	0.58–1.46	0.714															
O-level	0.87	0.60–1.24	0.436															
A-level	0.68	0.46–0.99	0.047															
degree	0.50	0.32–0.79	0.003															
*paternal education (ref.: none/CSE)*																		
vocational	0.85	0.55–1.31	0.466															
O-level	0.76	0.54–1.07	0.114															
A-level	0.76	0.55–1.05	0.099															
degree	0.59	0.40–0.86	0.007															
*home ownership status (ref.: rented)*																		
mortgaged	0.74	0.48–1.13	0.167															
owned	0.55	0.31–0.97	0.041															
*financial difficulties (ref.: none)*																		
some	1.12	0.89–1.41	0.328															
many	1.02	0.72–1.44	0.925															
maternal age at menarche				0.95	0.86–1.05	0.313												
maternal age at first pregnancy				0.80	0.72–0.90	<0.001												
mother had sex when less than 16				1.72	1.28–2.33	0.001												
paternal age at index pregnancy				0.96	0.86–1.07	0.462												
maternal parenting score							1.00	0.90–1.11	0.998									
paternal parenting score							1.02	0.91–1.13	0.786									
father absence before the age of 10 (ref.: present)							1.87	1.40–2.49	<0.001									
Index of Multiple Deprivation 2010										1.14	1.03–1.28	0.015						
*Big Five personality dimensions*																		
extraversion													2.19	1.92–2.50	<0.001			
agreeableness													0.90	0.79–1.02	0.090			
conscientiousness													0.83	0.73–0.94	0.004			
emotional stability													0.80	0.71–0.91	0.001			
openness													0.92	0.81–1.04	0.198			
pubertal development																1.72	1.38–2.16	<0.001
	*Var.*	*Prop.*		*Var.*	*Prop.*		*Var.*	*Prop.*		*Var.*	*Prop.*		*Var.*	*Prop.*		*Var.*	*Prop.*	
explained variance	0.377	0.076		0.329	0.069		0.256	0.053		0.222	0.046		0.781	0.154		0.255	0.053	
residual variance	4.557	0.924		4.457	0.931		4.802	0.947		4.576	0.954		4.286	0.846		4.557	0.947	
school residual variance	0.064	0.013		0.079	0.016		0.120	0.025		0.117	0.024		0.130	0.026		0.145	0.030	
neighbourhood residual variance	0.052	0.011		0.054	0.011		0.074	0.015		0.075	0.016		0.110	0.022		0.069	0.014	
friends residual variance	1.151	0.233		1.035	0.216		1.062	0.221		1.095	0.228		0.756	0.149		1.053	0.219	
individual residual variance	3.290	0.667		3.290	0.687		3.290	0.685		3.290	0.686		3.290	0.649		3.290	0.684	
total variance	4.934	1		4.786	1		4.802	1		4.798	1		5.066	1		4.812	1	

Household SES was negatively associated with the probability of a young person having had sex. Young people were less likely to be sexually experienced if their parents were more educated (OR = 0.50, *p* = 0.003 for maternal degree versus no educational qualification or CSE; OR = 0.59, p = 0.01 for paternal degree versus no educational qualification or CSE) or if living in owned rather than rented accommodation (OR = 0.55, *p* = 0.04). Mother-reported financial difficulties did not independently predict whether adolescents had had sex. The SES model explained 7.6% of the variance, but the residual intraclass correlation for friendship networks was hardly affected by the addition of indicators of household SES (rICC = 0.233 versus 0.250 for the full clustering-only model).

Two parental life history variables were significant in the unadjusted parental life history pace model. Adolescents whose mother was older at first pregnancy were less likely to have had sex (OR = 0.80, *p* < 0.001). Those whose mother had an early sexual debut (less than 16) were more likely to have had sex (OR = 1.72, *p* = 0.001). Maternal age at menarche and paternal age at index pregnancy did not add predictive power to the model. The parental life history pace model explained 6.9% of the variance; the residual intraclass correlation for friendship networks was somewhat reduced (rICC = 0.216).

Parenting scores were not significantly associated with sexual experience in late adolescence, but adolescents who grew up in a father-absent household were more likely to have had sexual intercourse (OR = 1.87, *p* < 0.001). Higher levels of neighbourhood deprivation were associated with an increased probability of my measure of sexual experience (OR = 1.14, *p* = 0.02). The neighbourhood deprivation model explained 4.6% of the variance; neighbourhood deprivation did not lead to substantial reduction of residual clustering at the friendship-network level (rICC = 0.228).

Three of the Big Five personality factors were significant predictors of whether an adolescent had had sex by 17.5 years of age. Adolescents who score higher on extraversion (OR = 2.19, *p* < 0.001), lower on conscientiousness (OR = 0.83, *p* = 0.004) and lower on emotional stability (OR = 0.80, *p* = 0.001) were more likely to have had sex. The unadjusted personality model, with age and sex, explained 15.4% of the variance. Out of all the (sets of) predictors, only personality traits appeared able to account for part of the clustering at the level of friendship networks. Adding personality reduced the residual intraclass correlation at the level of friendship networks from 0.250 to 0.149, a finding that suggests that some of the similarity between friends in sexual activity may be due to similarity in personality traits.

#### 3.3.2. Full model

**Figure 1.**
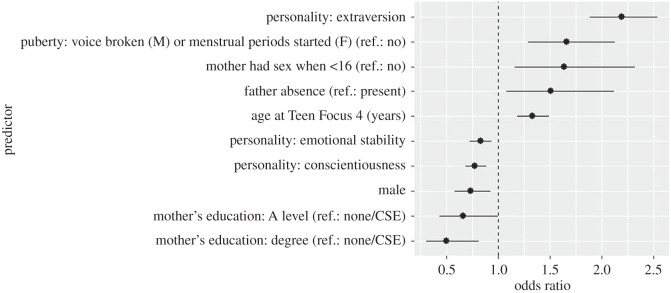
Odds ratios with 95% confidence intervals for significant predictors of experience with sexual intercourse. For continuous predictors the OR corresponds to a 1 s.d. increase; for categorical variables, the OR compares membership of a category to a reference category.

In the full, adjusted model (table 5; see figure 1 for visual impression of effect sizes), parental education was still a significant predictor (OR = 0.50, *p* = 0.01 for maternal degree versus no educational qualification or CSE), although paternal degree (*B* = 0.68, *p* = 0.07) just lost significance at the 5% level, while the coefficients for home ownership status were substantially smaller and no longer significant (OR = 0.82, *p* = 0.52 for owned versus rented). Maternal age at first pregnancy is no longer significant (OR = 0.93, *p* = 0.25). Another notable difference between the full and unadjusted models is that the estimate of the coefficient for the Index of Multiple Deprivation 2010 was strongly reduced and no longer significant (OR = 1.02, *p* = 0.73). Extraversion (OR = 2.19, *p* < 0.001), conscientiousness (OR = 0.78, *p* < 0.001) and emotional stability (OR = 0.83, *p* = 0.005) remained significant predictors; agreeableness (OR = 0.92, *p* = 0.25) and openness (OR = 0.97, *p* = 0.67) did not predict sexual behaviour.

**Table 5.** Model results for a combined household socioeconomic status + parental life histories + parental investment + neighbourhood deprivation + personality model; the same model plus respondent's education.

	all predictors except adolescent education			all predictors including adolescent education		
parameter	OR	95% CI	*p*-value	OR	95% CI	*p*-value
age at Teen Focus 4	1.33	1.19–1.49	<0.001	1.34	1.19–1.50	<0.001
sex	0.74	0.58–0.93	0.012	0.64	0.50–0.82	<0.001
*maternal education (ref.: none/CSE)*						
vocational	0.93	0.57–1.53	0.784	0.93	0.56–1.53	0.768
O-level	0.83	0.57–1.22	0.354	0.87	0.59–1.28	0.467
A-level	0.66	0.44–1.00	0.048	0.75	0.49–1.14	0.176
degree	0.50	0.31–0.82	0.006	0.63	0.39–1.04	0.071
*paternal education (ref.: none/CSE)*						
vocational	0.85	0.54–1.34	0.481	0.83	0.53–1.31	0.431
O-level	0.88	0.61–1.26	0.478	0.93	0.64–1.34	0.690
A-level	0.82	0.58–1.16	0.257	0.90	0.63–1.27	0.535
degree	0.68	0.45–1.02	0.066	0.83	0.55–1.26	0.387
*home ownership status (ref.: rented)*						
mortgaged	0.97	0.61–1.54	0.882	1.06	0.66–1.71	0.796
owned	0.82	0.45–1.50	0.522	0.93	0.51–1.72	0.823
*financial difficulties (ref.: none)*						
some	1.05	0.83–1.32	0.704	1.02	0.80–1.29	0.894
many	0.90	0.61–1.33	0.609	0.89	0.60–1.31	0.546
maternal age at menarche	0.97	0.86–1.09	0.602	0.96	0.85–1.08	0.495
maternal age at first pregnancy	0.93	0.82–1.05	0.254	0.94	0.83–1.07	0.330
mother had sex when less than 16	1.64	1.16–2.32	0.005	1.63	1.15–2.31	0.006
paternal age at index pregnancy	1.00	0.88–1.12	0.932	1.00	0.89–1.13	0.992
maternal parenting score	1.00	0.90–1.12	0.959	1.02	0.91–1.14	0.769
paternal parenting score	1.07	0.95–1.21	0.257	1.08	0.96–1.21	0.226
father absence (ref.: present)	1.51	1.08–2.12	0.016	1.46	1.04–2.04	0.029
Index of Multiple Deprivation 2010	1.02	0.90–1.15	0.728	0.98	0.87–1.11	0.728
GCSE results (ref.: no A or A*)						
one				0.68	0.49–0.96	0.027
two				0.49	0.32–0.74	0.001
three				0.56	0.35–0.89	0.016
four, five or six				0.44	0.31–0.63	<0.001
seven or more				0.31	0.22–0.45	<0.001
*Big Five personality dimensions*						
extraversion	2.19	1.89–2.54	<0.001	2.13	1.83–2.47	<0.001
agreeableness	0.92	0.81–1.06	0.251	0.95	0.83–1.09	0.427
conscientiousness	0.78	0.69–0.89	<0.001	0.80	0.70–0.91	0.001
emotional stability	0.83	0.73–0.94	0.005	0.84	0.73–0.96	0.01
openness	0.97	0.85–1.11	0.670	1.08	0.94–1.24	0.273
pubertal development	1.66	1.29–2.13	<0.001	1.69	1.31–2.17	<0.001
			*proportion of*			*proportion of*
	*variance*	*95% CI*	*total variance*	*variance*	*95% CI*	*total variance*
explained variance	1.193		0.209	1.396		0.236
residual variance	4.522		0.791	4.526		0.764
school residual variance	0.079	−0.02–0.19	0.014	0.051	−0.04–0.14	0.009
neighbourhood residual variance	0.078	−0.08–0.24	0.014	0.075	−0.09–0.24	0.013
friends residual variance	1.075	0.35–1.80	0.188	1.110	0.35–1.87	0.187
individual residual variance	3.290		0.576	3.290		0.556
total variance	5.715		1.000	5.922		1.000

Compared with the multiple classification model without substantive predictors (apart from age and sex), the residual ICC of schools decreased from 0.023 to 0.014, that of neighbourhoods from 0.010 to 0.014, and that of friendship networks by nearly roughly a quarter, from 0.250 to 0.188. The proportion of explained variance for the full model, without respondent's educational achievement, was 0.209.

#### 3.3.3. Full model + adolescent educational achievement

Because, like sexual experience, adolescents' educational achievement possibly reflects their life history pace (§1.1.2), it may represent a correlated outcome rather than a (potential) causal factor. Moreover, adolescent education could be on a causal pathway between parental education and sexual behaviour. For the purpose of clarity, we therefore ran the full model both without (results above) and with adolescent educational achievement. The higher the number of A or A* results a respondent achieved, the lower was the probability of him or her having had sex by 17.5 years of age (table 5). Adding educational achievement increased the explained variance, as a proportion of total variance, from 0.209 to 0.236. Adding the respondent's educational achievement to the full model markedly reduced the estimates of the effects of maternal and paternal education, neither of which retained statistical significance. The proportion of explained variance for an education-only model (with age and sex; electronic supplementary material, table S3) was 0.104.

## 4. Discussion

### 4.1. Main findings

In a sample of contemporary British adolescents, experience of sexual behaviour by 17.5 years clustered at the level of friendship networks but not in schools or neighbourhoods. This clustering among friends was not due, for the most part, to clustering of life history factors that predict sexual behaviour. Friendship formation based on similarities in personality traits did appear to explain about a quarter of the clustering of experience of sexual activity among friends. Life history predictors such as SES and father absence before the age of 10 did account for some of the variation found in adolescent experience of sexual behaviour but no evidence was found for effects of parental care or neighbourhood deprivation, counter to predictions of some life history models of adolescent development.

Friends are far more similar than non-friend age peers going to the same school or living in the same neighbourhood. This suggests that differences between schools and neighbourhoods, in this sample, have little impact on adolescent experience of sexual activity by age 17.5. Nor do schools and neighbourhoods appear to possess differing normative climates resulting in different levels of sexual activity. These results are consistent with a multiplier effect for individual-level interventions as behaviours may cascade through friendship networks [78], although this would require that similarity of friends is at least partly due to social influence, something we were unable to test directly.

In contrast with expectations based on life history theory, neighbourhood deprivation did not predict experience of sexual activity, consistent with results of previous multisystem studies of risky adolescent sexual behaviour [62–64], which did not find measures of perceived neighbourhood quality or exposure to violence to show the associations predicted by life history theory. On the other hand, other studies (e.g. [2,40,41]) did find that ecological context at the neighbourhood level—socioeconomic deprivation and life expectancy—predicted age at first birth. These differences might be explained by the fact that the studies mentioned used age at first birth as an outcome rather than experience with sexual intercourse. Moreover, the neighbourhoods in Wilson & Daly's study [40] showed an extraordinary range in homicide levels and (homicide-adjusted) life expectancy, which might result in more pronounced life history effects. It should be noted, however, that Nettle [2] did not control for household SES [2]; and the study by Wilson & Daly [40] was performed entirely at the neighbourhood level. A large study from Northern Ireland that did look at both individual and neighbourhood levels found individual SES to be by far the largest predictor of age at first birth, but neighbourhood characteristics to have small additional effects [41].

The fact that we found no school-level clustering to speak of is possibly due to our definition of the school classification, which was simply based on a school identifier. A school class classification might have revealed clustering consistent with school-peer influences (cf. [78,75,82]), but note that class composition differs by subject in UK secondary schools, making school class less meaningful than in primary schools.

Personality was a strong predictor of experience of sexual intercourse, in line with existing studies on sexual behaviour [5,53–55] and reproductive success [56,105]. Our results suggest that a sizeable part, around a quarter in the sample, of the similarity of sexual behaviour among friends is due to clustering of personality traits in friendship networks. Thus, studies that do not take similarity in personality into account may be overestimating the effect of (descriptive) peer norms on adolescent sexual behaviour.

Our finding that extraverted individuals are more likely to have had sex is consistent with previous findings on extraversion and risky sexual behaviour [5,53–55], and fits with Nettle's suggestion that extraversion evolved as a life history strategy premised on high mating success [5,55], characterized by an early sexual debut, unstable pair-bonds and a high number of sexual partners. The fact that lower conscientiousness predicted a higher probability of having had sex is also in line with previous studies [53,106], and the general short-term orientation of low-conscientiousness individuals [107]. Likewise, the negative relationship between emotional stability and sexual intercourse is in line with the existing empirical literature [53]. Unlike several previous studies (reviewed in [53]), which found a negative association between agreeableness and risky sexual behaviour, we did not find an effect of agreeableness on the probability of having had sex. This difference may be due to the nature of our outcome measure, which did not measure *risky* sexual behaviour. Moreover, agreeableness is a desirable attribute in a potential partner which, by increasing opportunities for entering romantic relationships, could counteract any negative effect agreeableness might have on the tendency to engage in sexual intercourse.

While we cannot test for it directly, our results indicate more scope for social transmission of sexual norms and attitudes between members of a friendship network, than between (non-friend) peers in school or neighbourhoods. And while our results are consistent with the notion of peer groups as key socialization sources [108], they cannot be used to infer a relatively minor role of parents-as-socializers [108], as we did not test the latter hypothesis. Apart from social influence, assortment on the outcome, or similarity in unmeasured predictors thereof, could also account for clustering. The negative association of sexual experience with educational achievement suggests this may in part reflect trade-offs made by adolescents in how they invest efforts between education and social and sexual behaviour. Friends (or their parents) may be influential in making such decisions [109] or indeed help each other achieve social or educational goals.

### 4.2. Strengths and limitations

This study has a number of notable strengths. Most importantly, it includes friendship networks, neighbourhoods and schools in a multiple classification modelling framework, allowing for simultaneous estimation of social clustering at each level while taking the others into account. The inclusion of friendship networks as an additional classification in a multiple classification multilevel model [95], alongside schools and neighbourhoods, is a novel feature. We were also able to include a wide range of theoretically interesting predictors, such as neighbourhood deprivation and personality, because of ALSPAC's comprehensiveness. The use of ALSPAC also ensured the availability of a relatively large sample.

Several limitations should be kept in mind when interpreting the results reported here. As noted earlier, our analysis sample is skewed toward families of higher SES in less deprived neighbourhoods (see electronic supplementary material, S1). It is possible that the effects of life history predictors largely or exclusively manifest themselves at their extremes. Perhaps behavioural life history traits may be found to cluster much less in friendship networks, after accounting for the effects of life history predictors, in more extreme (e.g. very harsh) settings, where environmental conditions might predominate in line with life history predictions. Future studies could address this sort of question by considering whether social clustering patterns vary across contexts.

If closer friends, who are more likely to reciprocate friendship nominations, are more influential [110], then the inclusion of non-reciprocated friendship nominations would bias (downwards) our estimate of friend similarity. When a study based on data from Add Health explicitly addressed this question in an investigation of peer influence sexual activity, it found no evidence for an interaction between the sexual activity of the closest friend and whether or not the friend nomination was reciprocated [77]. We also included individuals in the same friendship network if they share a friend, which might dilute clustering.

### 4.3. Conclusion

In a sample of British adolescents, experience with sexual intercourse clustered in adolescent friendship networks, but not in neighbourhoods or schools. Thus, the clustering we found in friendship networks was not due to friends experiencing similar socio-ecological circumstances at the level of neighbourhoods or schools. While life history predictors did explain some of the variation in sexual activity, they did not explain much social clustering, strongly suggesting that similarity of life history predictors among friends is not responsible for clustering of sexual experience in friendship networks. Instead, the social clustering of sexual behaviour among friends could be due to a tendency to associate with similar others, perhaps because of potential coordination benefits from similarity, or due to social transmission of norms of behaviour among friends, possibly reflecting conformism or prestige bias.

## Supplementary Material

Table S1. Comparison of baseline characteristics for analysis and attrition sample. The attrition sample consists of all individuals (singleton births) in the core ALSAC sample who were alive at 1 year but not included in the analysis sample.

## Data accessibility

Data used for this submission will be made available on request to the Executive (alspac-exec@bristol.ac.uk). The ALSPAC data management plan (available here: http://www.bristol.ac.uk/alspac/researchers/data-access/documents/alspac-data-management-plan.pdf) describes in detail the policy regarding data sharing, which is through a system of managed open access.
