# Supplementary material for: Life history factors, personality and the social clustering of sexual experience in adolescents
Source: R Soc Open Sci. 2016 Oct 5;3(10):160257. doi: 10.1098/rsos.160257 (PMC5098968; doi:10.1098/rsos.160257)
Supplement: Table S1. Comparison of baseline characteristics for analysis and attrition sample. The attrition sample consists of all individuals (singleton births) in the core ALSAC sample who were alive at 1 year but not included in the analysis sample. [file rsos160257supp1.docx]

**Table S1.** Comparison of baseline characteristics for analysis and attrition sample. The attrition sample consists of all individuals (singleton births) in the core ALSAC sample who were alive at 1 year but not included in the analysis sample.

| **Variables** | **Units *or* categories** | **Analysis sample** | **Attrition sample** | **Test for difference** |
| --- | --- | --- | --- | --- |
| Maternal education | None/CSE | 350 (12.4%) | 2,089 (22.5%) | X^2^ = 180.6, p < 0.001 |
|  | Vocational | 230 (8.1%) | 966 (10.4%) |  |
|  | O-levels | 1,050 (37.2%) | 3,135 (33.8%) |  |
|  | A-levels | 745 (26.4%) | 1,983 (21.4%) |  |
|  | Degree | 451 (16.0%) | 1,103 (11.9%) |  |
|  | Total | 2,826 | 9,276 |  |
| Paternal education | None/CSE | 509 (18.5%) | 2,529 (28.5%) | X^2^ = 115.0, p < 0.001 |
|  | Vocational | 235 (8.5%) | 750 (8.5%) |  |
|  | O-levels | 640 (23.0%) | 1,822 (20.5%) |  |
|  | A-levels | 786 (28.5%) | 2,247 (25.3%) |  |
|  | Degree | 584 (21.0%) | 1,526 (17.2%) |  |
|  | Total | 2,754 | 8,874 |  |
| Home ownership (at pregnancy) | Mortgaged | 2,357 (85.6%) | 6,674 (70.3%) | X^2^ = 273.8, p < 0.001 |
|  | Owned | 61 (2.2%) | 217 (2.3%) |  |
|  | Rented | 337 (12.2%) | 2,608 (27.5%) |  |
|  | Total | 2,755 | 9,499 |  |
| Index of Multiple Deprivation (ward at birth) | Composite score | Median = 14.2; range = 3.9 – 66.8 | 18.3; range = 3.9 – 66.8 | Median test: X^2^ = 273.8, p < 0.001 |
|  | Total | 2,696 | 10,048 |  |
| Maternal age at first pregnancy | Years | 25.7 (4.8) | 24.0 (5.0) | t = 16.9, p <0.001 |
|  | Total | 2,845 | 9,970 |  |
| Education (GCSE results): number of A or A* results | 0 | 1,179 (41.0%) | 5,764 (67.2%) | X^2^ = 682.7, p < 0.001 |
|  | 1 | 382 (13.3%) | 830 (9.7%) |  |
|  | 2 | 228 (8.0%) | 421 (4.9%) |  |
|  | 3 | 170 (5.9%) | 290 (3.4%) |  |
|  | 4-6 | 393 (14.0%) | 634 (7.4%) |  |
|  | 7-14 | 525 (18.0%) | 637 (7.4%) |  |
|  | Total | 2,877 | 8,576 |  |

*
